# Quantification of Cervical Cord Cross-Sectional Area: Which Acquisition, Vertebra Level, and Analysis Software? A Multicenter Repeatability Study on a Traveling Healthy Volunteer

**DOI:** 10.3389/fneur.2021.693333

**Published:** 2021-08-04

**Authors:** Carsten Lukas, Barbara Bellenberg, Ferran Prados, Paola Valsasina, Katrin Parmar, Iman Brouwer, Deborah Pareto, Àlex Rovira, Jaume Sastre-Garriga, Claudia A. M. Gandini Wheeler-Kingshott, Ludwig Kappos, Maria A. Rocca, Massimo Filippi, Marios Yiannakas, Frederik Barkhof, Hugo Vrenken

**Affiliations:** ^1^Institute of Neuroradiology, St. Josef Hospital, Ruhr-University Bochum, Bochum, Germany; ^2^Department of Medical Physics and Biomedical Engineering, Centre for Medical Image Computing (CMIC), University College London, London, United Kingdom; ^3^Queen Square Multiple Sclerosis Centre, UCL Queen Square Institute of Neurology, University College London, London, United Kingdom; ^4^e-Health Centre, Universitat Oberta de Catalunya, Barcelona, Spain; ^5^Neuroimaging Research Unit, Division of Neuroscience, IRCCS San Raffaele Scientific Institute, Milan, Italy; ^6^Neurological Clinic and Policlinic, Department of Medicine, University Hospital Basel, Basel, Switzerland; ^7^Department of Radiology and Nuclear Medicine, Multiple Sclerosis Center Amsterdam, Amsterdam Neuroscience Amsterdam University Medical Centers (UMC), Vrije Universiteit Medical Center (VUmc), Amsterdam, Netherlands; ^8^Section of Neuroradiology, Department of Radiology, Hospital Universitari Vall d'Hebron, Barcelona, Spain; ^9^Department of Neurology–Neuroimmunology, Multiple Sclerosis Center of Catalonia (Cemcat), Hospital Universitari Vall d'Hebron, Barcelona, Spain; ^10^Department of Brain & Behavioral Sciences, University of Pavia, Pavia, Italy; ^11^Brain Connectivity Center, IRCCS Mondino Foundation, Pavia, Italy; ^12^Department of Biomedical Engineering, University of Basel, Allschwig, Switzerland; ^13^Neurology Unit, IRCCS San Raffaele Scientific Institute, Milan, Italy; ^14^Vita-Salute San Raffaele University, Milan, Italy; ^15^Neurorehabilitation Unit, IRCCS San Raffaele Scientific Institute, Milan, Italy; ^16^Neurophysiology Service, IRCCS San Raffaele Scientific Institute, Milan, Italy

**Keywords:** spinal cord, cervical cord, atrophy, cross-sectional area, CSA, MRI, cord segmentation software, multiple sclerosis

## Abstract

**Background:** Considerable spinal cord (SC) atrophy occurs in multiple sclerosis (MS). While MRI-based techniques for SC cross-sectional area (CSA) quantification have improved over time, there is no common agreement on whether to measure at single vertebral levels or across larger regions and whether upper SC CSA can be reliably measured from brain images.

**Aim:** To compare in a multicenter setting three CSA measurement methods in terms of repeatability at different anatomical levels. To analyze the agreement between measurements performed on the cervical cord and on brain MRI.

**Method:** One healthy volunteer was scanned three times on the same day in six sites (three scanner vendors) using a 3T MRI protocol including sagittal 3D T1-weighted imaging of the brain (covering the upper cervical cord) and of the SC. Images were analyzed using two semiautomated methods [NeuroQLab (NQL) and the Active Surface Model (ASM)] and the fully automated Spinal Cord Toolbox (SCT) on different vertebral levels (C1–C2; C2/3) on SC and brain images and the entire cervical cord (C1–C7) on SC images only.

**Results:** CSA estimates were significantly smaller using SCT compared to NQL and ASM (*p* < 0.001), regardless of the cord level. Inter-scanner repeatability was best in C1–C7: coefficients of variation for NQL, ASM, and SCT: 0.4, 0.6, and 1.0%, respectively. CSAs estimated in brain MRI were slightly lower than in SC MRI (all *p* ≤ 0.006 at the C1–C2 level). Despite protocol harmonization between the centers with regard to image resolution and use of high-contrast 3D T1-weighted sequences, the variability of CSA was partly scanner dependent probably due to differences in scanner geometry, coil design, and details of the MRI parameter settings.

**Conclusion:** For CSA quantification, dedicated isotropic SC MRI should be acquired, which yielded best repeatability in the entire cervical cord. In the upper part of the cervical cord, use of brain MRI scans entailed only a minor loss of CSA repeatability compared to SC MRI. Due to systematic differences between scanners and the CSA quantification software, both should be kept constant within a study. The MRI dataset of this study is available publicly to test new analysis approaches.

## Introduction

Spinal cord (SC) atrophy assessment in neurological diseases such as multiple sclerosis (MS) has gained important attention over the past years (1–3). Techniques for measuring SC volume or cross-sectional area (CSA) on the basis of magnetic resonance imaging (MRI) have improved in reliability by the use of semiautomated and fully automated techniques largely replacing time-consuming manual outlining of the cord (4, 5). Some of these techniques have already been established in the field of MS, analyzing large cohorts of patients (6–12).

Most of the studies concentrated on CSA measurements in the upper cervical regions including individual levels at the C2/C3 vertebra (6) or larger volumetric regions (9, 13), while a few studies used quantification of the entire cervical cord (5, 10). Furthermore, it has been shown that cervical SC atrophy can also be quantified using 3D T1-weighted (3D T1w) brain MRI scans that include the upper cervical cord region as well as by using T2-weighted and other MRI sequences and combinations of image contrast (8, 13–15). For a current meta-analysis of MRI-based SC quantification studies in MS, see Casserly et al. (2). Still, the choice of the specific cord level can influence results due to physiological variability in the cord area or disease-related patterns of atrophy along the cervical cord (10, 16). Moreover, recent comparative studies have shown that the CSA estimates can differ systematically between the various software methods (11, 17). Hence, harmonization of analyses remains challenging. Multicentric studies performed under harmonized conditions have been increasingly used to explore systematic differences caused by MRI scanners, image acquisition methods (pulse sequences and parameter settings), and volumetric analyses (11, 18–21). But so far, no common agreement on methods for SC atrophy quantification has been established.

In this multicenter single-subject study, we aim at identifying a common and reliable regional level of CSA measurement by comparing different software methods with respect to variability and systematic differences at different cervical cord levels using brain and cord MRI. We intend to widen the scope of previous comparable studies by including other software tools, more SC levels, and a larger number of different scanners (15, 22). The study design reflects a real-world multicenter scenario with harmonization of the MRI protocol for sagittal isotropic 3D T1w brain and cervical cord imaging with respect to image resolution and contrast, but without the exact specification of the sequence design and timing. We quantitatively compare three established software methods for CSA assessment [NeuroQLab (NQL), Active Surface Model (ASM), and Spinal Cord Toolbox (SCT)] in terms of reliability at three different cervical cord levels: at the upper SC at the vertebral levels C1–C2, at the level of the vertebral disc C2/3, and at the entire cervical SC (C1–C7). In this process, we compare high-resolution 3D T1w MRI of the brain and cervical SC of six European centers, one set for covering the whole brain and the upper part of the cervical SC and one specifically optimized for the SC.

In addition, the current work provides a publicly available reference MRI dataset to the research community, containing single-subject, multicenter, repeated volumetric acquisitions of the brain and cervical SC in a healthy volunteer.

## Materials and Methods

### MRI

A single healthy volunteer (male, age 45 years at first scan) underwent multiple repeated MRI scans at six different European centers with high expertise in MRI for MS. The scans at a given site were acquired during a single visit. At all scanners, the time of examination was in the afternoon between 3:00 and 7:00 p.m., thus minimizing the effects of potential daytime-dependent fluctuations of the SC volume. The acquisitions at five of the centers took place between March 2015 and February 2016 and at center 6 in January 2017. Given the limited time period and the age of the healthy volunteer, we assumed that the true CSA could be considered stable between the scans given the slow cervical cord atrophy rate in healthy controls [e.g., (10)].

The participant gave written informed consent to take part in the repeated MRI acquisitions at different centers both for the use of the anonymized MRI data for scientific purposes within the scope of the present study and for sharing the data with the research community. At each site, the MRI acquisitions reported in the present study were conducted after signed informed consent of the volunteer and with the approval of ethics boards of the involved institutions.

#### MRI Acquisition

Scanning included a sagittal isotropic 3D T1w sequence of the brain covering the upper cervical cord including at least the C1–C3 vertebral levels, which was followed by acquisition of a sagittal isotropic 3D T1w sequence of the entire SC using combined neurovascular head and neck matrix coils. Both sequences were acquired three times with repositioning of the subject in the scanner between the second and third examination in order to incorporate potential effects of different positioning within the magnet. Image acquisition followed a consent protocol within the MAGNIMS consortium, which was standardized for geometry requiring isotropic image resolution of 1 × 1 × 1 mm^3^, and accepted magnetization-prepared, high-contrast 3D T1w gradient-echo imaging according to the local expertise and the specific software/hardware available in each of the participating centers. Thus, the study design was intended to reflect a real-world multicenter scenario without the exact specification of the sequence design and timing. All scans were performed using 3-Tesla scanners (one General Electric, three Philips, and two Siemens). All sites used the vendor-specific 3D distortion correction procedures to correct for non-linear gradient distortion effects (23). Detailed imaging parameters for each sequence at each site are provided in Table 1.

**Table 1:** Sequence parameters used with the 3-Tesla scanners at the participating centers.

**Site Nr**	**1** ** Milano**	**2** ** Bochum**	**3** ** Barcelona**	**4** ** London**	**5** ** Amsterdam**	**6** ** Basel**
Vendor/model	Philips Intera	Philips Achieva	Siemens TIM Trio	Philips Achieva	GE discovery MR750	Siemens Prisma
Magnet length/bore diameter (cm)	157/60	157/60	198/60	157/60	194/60	198/60
Coil type	SENSE-NV-16 ch.	SENSE-NV-16 ch.	Head-Neck array 16 ch.	SENSE-NV-16 ch.	HNS array 16 ch.	Head-Neck array 64 ch.
MRI date	03/2015	06/2015	12/2015	01/2016	02/2016	01/2017
MRI time	6:30 p.m.	3:00 p.m.	7:00 p.m.	4:00 p.m.	4:00 p.m.	4:30 p.m.
**Head MRI**						
Sequence	TFE	TFE	MPRAGE	TFE	FSPGR	MPRAGE
Number of Slices	204	180	192	180	172	176
Orientation	Sagittal	Sagittal	Sagittal	Sagittal	Sagittal	Sagittal
TR (ms)	7.1	10	2,300	6.8	7.8	1570
TE (ms)	3.2	4.6	3	3.1	3	2.48
TI (ms)	900	1,000	900	825	450	900
FA	9°	8°	9°	8°	12°	8°
Voxel size (mm)	1 × 1 × 1	1 × 1 × 1	1 × 1 × 1	1 × 1 × 1	0.98 × 0.98 × 1	1 × 1 × 1
Acquisition duration	8:32 min	5:44 min	5:01 min	6:32 min	7:07 min	4:06 min
**Cervical cord MRI**						
Sequence	TFE	TFE	MPRAGE	TFE	FSPGR	MPRAGE
Number of Slices	64	64	128	128	172	176
Orientation	Sagittal	Sagittal	Sagittal	Sagittal	Sagittal	Sagittal
TR (ms)	8	8	2,300	8	7.3	1,570
TE (ms)	3.5	3.5	3.26	3.7	3	2.48
TI (ms)	1,000	1,000	900	856	450	900
FA	8°	8°	9°	8°	15°	8°
Voxel Size (mm)	1 × 1 × 1	1 × 1 × 1	1 × 1 × 1	1 × 1 × 1	1 × 1 × 1	1 × 1 × 1
Acquisition duration	4:05 min	4:08 min	5:01 min	6:32 min	5:57 min	4:06 min

*TR, repetition time; TE, echo time; TI, inversion time; FA, flip angle; TFE, turbo field echo; MPRAGE, magnetization prepared rapid acquisition gradient echo; FSPGR, fast spoiled gradient recalled echo*.

*Site Numbers: 1 = Ospedale San Raffaele, Milano; 2 = Ruhr-University of Bochum, Bochum; 3 = Hospital Universitari Vall d'Hebron, Barcelona; 4 = University College London, London; 5 = VU University Medical Center, Amsterdam; 6 = Basel University Hospital, Basel*.

The anonymized MRI datasets of brain and cervical cord MRI used in this study are freely available for scientific research upon request to MAGNIMS (https://www.magnims.eu/magnims-cord-dataset/).

### Cross-Sectional Area Assessment

For CSA measurements, three different software methods were used that had previously been found to be reliable in large MS cohorts (7–9). The first approach consisted of the fully automated deformable model method PropSeg, freely available with the SCT (5) (version 4.1; https://sourceforge.net/projects/spinalcordtoolbox/). In addition to this fully automated approach, two semiautomated methods requiring manual interaction were chosen: the ASM, available with costs with the Jim Software package (JIM, v. 7.0, Xinapse Systems, Colchester, UK; www.xinapse.com) (4, 7) and the watershed-segmentation method available with NQL (Fraunhofer-Mevis, Bremen, Germany; license freely available for research purposes upon request from Fraunhofer-Mevis) (24, 25).

#### Spinal Cord Toolbox, “PropSeg”

SCT features specific segmentation tools for the SC. The segmentation algorithm PropSeg is based on an iterative propagation of a deformable model with an adaptive contrast mechanism (5, 26). Automated detection of the center of the SC is done by ellipse detection and information from the body symmetry, followed by propagating a tubular surface along the SC edge using deformable models. SCT has been applied in studies on MS patients and has been shown to be highly reproducible (17). We used SCT version 4.01 with default settings.

#### Active Surface Model

The ASM, which is implemented as the cord finder tool in the JIM software package, requires interactive marking of the center of the cord on a regular distance along several vertebral levels to be included in the analysis (4). Cord center line and cord outlines at each slice are then calculated using a segmentation algorithm with a steadily increasing refinement of the ASM. This allows a rapid semiautomated segmentation by measuring the cord CSA along the length of the extracted surface parameter. We used the cord finder tool included in JIM version 7.0, with the following settings: nominal cord diameter setting, 10 mm; number of shape coefficients, 18; order of longitudinal variation, 5. ASM has been shown to be highly reproducible, and the method has already been used in cross-sectional and in longitudinal MS studies (7, 10, 27, 28).

#### NeuroQLab

NQL requires the user first to interactively define the section of the cord to be analyzed by placing an oblique plane through the dataset, which runs through the upper and lower end of the section (24). This is aided by two perpendicular lines, which allow to align the section precisely to the specific vertebral bodies. This step is followed by a semiautomatic pre-segmentation using a watershed transformation of the pixel intensities. Subsequently, a fully automated model-based volume measurement is performed by fitting the intensity distribution of the pre-segmented input region using a Gaussian mixture model. The SC volume is modeled using Gaussian mixture of two tissue classes [spinal cord tissue and cerebrospinal fluid (CSF)] and a separate class representing partial volume voxels. The volume is calculated by summation of the SC tissue class volume and half of the volume of the partial volume class. The center line of the SC is calculated and used to determine the mean CSA by normalizing the measured volume to the section length. The operator can correct the final results interactively. NQL has been shown to be highly reproducible (24, 25), and the method has already been used in cross-sectional and in longitudinal studies of different neurological diseases including MS (8, 15, 22, 29–31).

#### Similarities and Differences Between the Software Methods Regarding Partial Volume Effects and Handling of Cord Curvature

The definition of the cord contour and rules for inclusion or exclusion of voxels located on the edge between cord and CSF may account for a substantial proportion of the segmented volume, given the limited image resolution and the small diameter of the SC. This aspect is handled differently between the software methods and may contribute to systematic differences between the CSA estimates generated with different algorithms (11, 17). While the SCT algorithm includes only voxels that are classified as “pure” cord tissue into the segmentation, without referring to partial volume effects at the margin of the cord contour, NQL takes voxels at the edge of the cord into account that are affected by partial volume effects by including pure cord tissue voxels and 50% of the partial-volume tissue class in the CSA calculation (17, 24). Similarly to NQL, the cord segmentation of ASM includes a fraction of those voxels subject to partial volume effects between the cord and the surrounding CSF because the cord surface definition in ASM is partly controlled by seeking high-intensity gradients (4).

Additionally, effects of the cervical cord curvature are treated differently between NQL and ASM or SCT. While ASM and SCT are optimized with regard to variations of the cord curvature (4, 5), NQL quantifies the cord volume between two parallel oblique planes and uses the center line merely for calculation of the mean cord area from the segmented volume (24). Thus, the CSA estimations by NQL might differ from the corresponding ASM or SCT results depending on the degree of curvature of the SC or the exact choice of the cord section to be analyzed.

#### Cross-Sectional Area Measurements at Different Cord Levels

**Figure 1:**
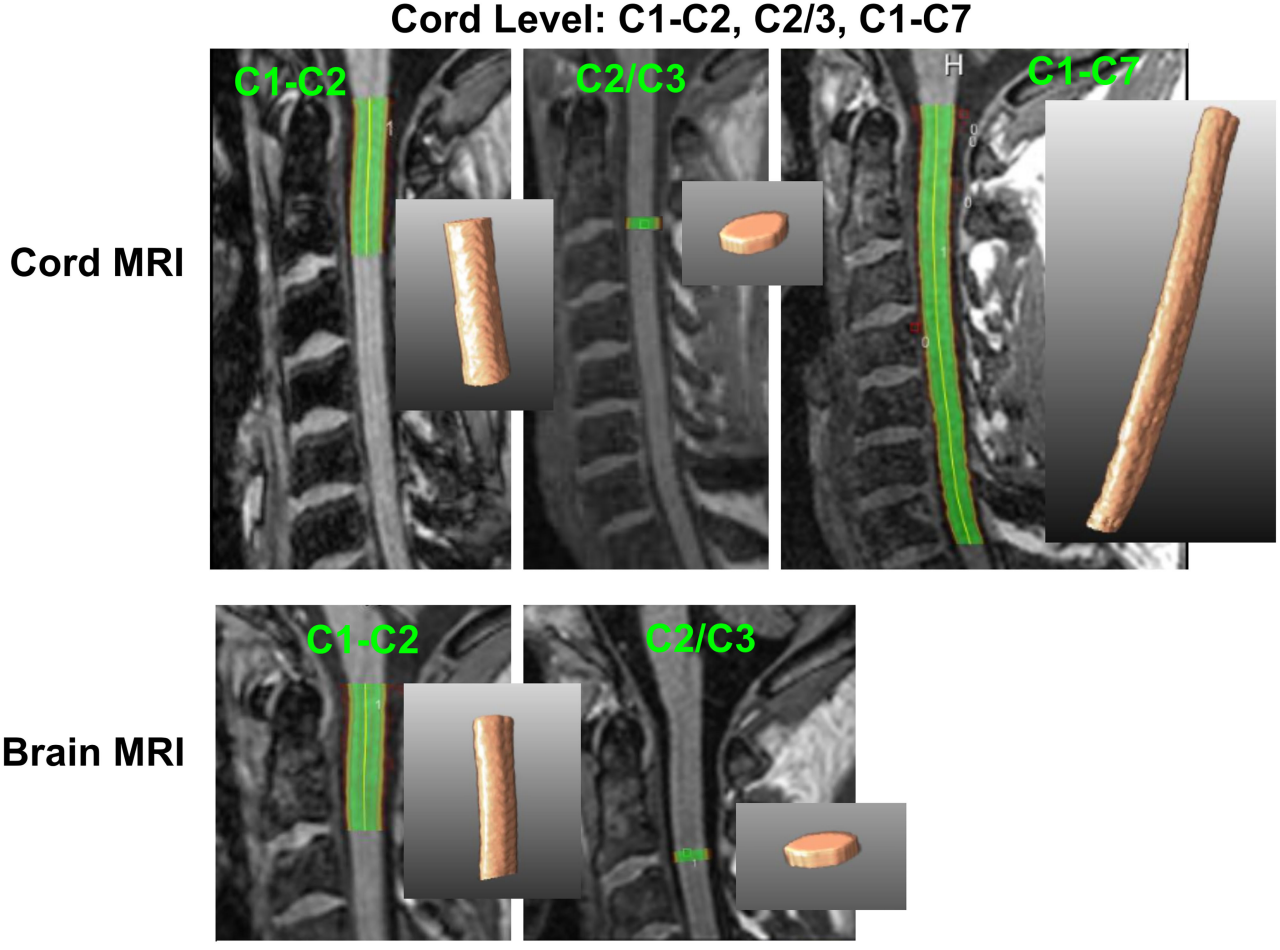
Definition of cervical cord level measurements on brain and cord MRI. C1–C2, upper cervical cord between C1 and C2 vertebra; C2/3, 3-mm slab at the C2/C3 disc level; C1–C7, entire cervical cord between the C1 and C7 vertebral level. 2D and 3D segmentations based on NQL are shown as examples.

CSA measurements were performed in different sections of the cervical cord, which were defined by anatomical markers. In all measurement setups, CSA represented the mean cross-sectional cord area within the chosen cord segment. Sections were chosen according to previously published procedures (7, 17, 25) and to match the procedural performance of the included software methods. For both head and SC acquisitions, CSA was measured at three different cord sections: at the level C1–C2, across the entire cervical cord from C1 to C7, and at the level of the intervertebral disc between C2 and C3 within a single slab of 3-mm thickness. Figure 1 illustrates which levels were investigated.

In the semiautomated methods (ASM and NQL), the C1–C2 and the C1–C7 sections were manually defined using the top of the dens and the endplate of the corresponding caudal vertebra (C3 or C7) as anatomical references for the upper and lower boundaries, respectively (7, 15, 25). The C2/C3 sections in ASM and NQL were manually defined by marking the level of the bottom of the C2 vertebral body as the upper boundary and including a 3-mm section caudally.

In SCT, the C1–C2 and C1–C7 sections were defined according to the automated vertebral labeling of the cord, which is part of the SCT algorithms (17). Since calculation of CSA in an arbitrary cord section was not provided in SCT, we determined CSA at the C2/C3 level based on the quantitative output reports of the software: the most caudal slice that was assigned to the C2 vertebra was manually determined from the SCT output, and the averaged CSA of three caudally adjacent slices (slice thickness 1 mm) was calculated.

The results of all methods were visually inspected for segmentation errors, false vertebra labeling (in case of SCT), or image artifacts. Two SCT measurements were excluded from the analyses due to erroneous segmentations. The C1–C7 CSA measurements of one center (site 6) were corrupted by infolding artifacts of the shoulders. We excluded these C1–C7 CSA estimates for all software methods. Only for this center, SCT segmentations in the C1–C2 and C2/3 sections were initiated by choosing the C2/C3 vertebral disc as a starting point for the segmentation to avoid PropSeg starting in the (lower) part of the image that contains image artifacts.

### Image Contrast Assessments

To compare contrast-to-noise ratios of cord tissue to CSF between the centers, the mean and standard deviations of signal intensities within regions of interest (ROI) were determined in the T1w MRI images. We placed ROI in the cervical cord at the C1–C2 level, the C2/3 level, the C5–C7 vertebral level, and in regions of the adjacent CSF using standard diagnostic image viewer tools. For this purpose, we used ovoid size-adapted contours placed well within the cord or CSF, each at the height of the level in question. The size of the ROIs was adjusted to cover as large an area as possible excluding the interface between cord tissue and CSF. For the C1–C2 cord level, the corresponding CSF ROI was placed within the cerebellomedullaris cistern, while for the C2/C3 and C5–C7 cord levels, the corresponding CSF ROIs were positioned in adjacent areas between the cord and the vertebral body (Details are shown in Supplementary Figure 1 in the electronic supplement.). For the ROIs at the C1–C2 and C2/C3 levels (brain and cord MRI) and the C5–C7 level (cord MRI only), we calculated the contrast-to-noise ratio per unit of time (CNR_UT_) between cord and CSF, controlling for differences between sites regarding the acquisition duration of the T1w sequences (32):

$$\begin{array}{l} \text{CN}{\text{R}}_{\text{UT}}\left(\text{cord to CSF}\right)=\frac{\left(\text{Signal}\left(\text{cord}\right)\;-\text{ Signal}\left(\text{CSF}\right)\right)}{\text{mean SD }\left(\text{cord},\text{ CSF}\right)}.\;\frac{1}{\sqrt{\text{t}}} \end{array}$$

where signal is the mean image intensity within the ROI, SD is the standard deviation, and t is the acquisition duration of MRI sequence (min).

### Statistical Analysis

Statistical analyses were performed using the software package SPSS (IBM, SPSS V. 25). Results were considered statistically significant when associated with *p* < 0.05.

#### Mean Maximum Observed Difference and Repeatability of CSA

To describe the smallest difference between two CSA measurements that could be detected in this multicenter setting with different scanners [named mean maximum observed difference (MMOD)], we determined the maximum of the absolute differences between the CSA results of the three repeated scans and averaged it across all centers (avg. max. abs. Δ_scans_). The MMOD was calculated separately for each software method and cord level in brain and SC MRI.

Additionally, the repeatability of the CSA measurements using the different software methods, cord levels, and brain or SC MRI was assessed by calculating the coefficient of variation (CV) of the three repeated scans using the formula, standard deviation_repetitions_/mean, and averaging the results over all centers.

Since the requirements of normal distribution and equality of variances of CSA within the group variables software methods, cord levels, and acquisition type (brain or SC MRI) were not met, we used non-parametric tests throughout the statistical analyses.

We checked for within-subject differences between the scan repetitions by using Wilcoxon signed rank tests for paired samples for the comparison of scan no. 1 with scan no. 2 (simple repetition) and scan no. 2 to scan no. 3 (repetition after repositioning of the healthy volunteer). By testing separately for each software method and cord level in brain and SC MRI, we detected no significant differences between the scan repetitions. Therefore, we did not include the scan repetition number as a factor in further statistical analyses. Given our atypical study design with only one subject, we rather handled the scans as independent measurements.

#### CSA Differences Between Methods

We assessed group differences of CSA between the software methods separately for MRI acquisition type (brain or cord) and cervical cord level (C1–C2, C2/3, C1–C7) by using Kruskal–Wallis tests. *Post-hoc* pairwise comparisons included Dunn's *post-hoc* tests with Bonferroni adjustment for multiple comparisons. In these analyses, the CSA estimates were aggregated across centers and scan repetitions.

#### Differences Between Brain and Cord MRI

Group differences between brain and cord MRI of CSA and group differences of the CV of CSA were assessed separately for the software methods and the C1–C2 or C2/3 level using Wilcoxon signed rank tests for paired samples. Therein, the CSA estimates were aggregated across centers and scan repetitions.

#### Between-Center Agreement

We investigated differences of CSA between sites at the C1–C2 or C2/3 cord levels separately for each software method while aggregating measurements of brain and cord MRI (*N* = 6 measurements each). We applied Kruskal–Wallis tests with *post-hoc* comparisons between pairs of sites adjusted for multiple comparisons with Dunn's *post-hoc* tests and Bonferroni adjustment.

#### Intensity and Contrast Assessment

We assessed group differences of the contrast-to-noise ratio of cord and CSF between the centers with Kruskal–Wallis rank tests. *Post-hoc* pairwise comparisons used Dunn's *post-hoc* tests with Bonferroni adjustment for multiple comparisons.

## Results

### Image Quality and Contrast in Different MRI Scanners

**Figure 2:**
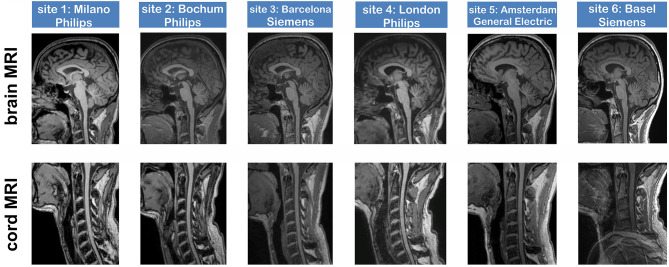
Image quality of 3D T1-weighted brain and spinal cord sequences at six 3-Tesla MRI scanners, acquired in the same healthy volunteer at all sites (age 45 years at first examination; male).

In total, 36 datasets, respectively, 18 pairs of brain (covering the upper cervical SC) and dedicated SC scans, were acquired in six centers using 3-Tesla MRI scanners of different vendors (Table 1). The SC MRI acquisition of site 6 included infolding artifacts in the caudal part of the images, so intensity measurements in these areas were omitted for site 6. Apart from that, visual inspection showed similar image quality and typical contrast settings of the sagittal, isotropic T1w sequences of brain and cervical cord across sites (Figure 2); however, intensity measurements (for cord imaging and brain imaging at the C1–C2 and C2/3 levels and for cord imaging at the lower vertebral levels C5–C7) revealed in all centers decreasing signal intensity toward the caudal parts of the images when comparing the different levels (Table 2). Moreover, the achieved contrast-to-noise ratio per unit of time between cord and CSF (CNR_UT_) differed significantly between the centers. When comparing the cord levels within each scanner, CNR_UT_ was similar between the C1–C3 and C2/3 cord levels in brain and cord MRI, while there was lower CNR_UT_ in the caudal parts of the images (C3–C5 cord level). In particular, for the dedicated cord acquisitions, the CNR_UT_ was higher in the Philips scanners (nr. 1, 2, 4) than that in the Siemens and GE scanners. Details are shown in Table 2.

**Table 2:** Region of interest-based cord signal and contrast-to-noise ratio per unit of time (CNR_UT_) of cord relative to CSF at different cervical cord levels compared for brain and cervical cord MRI scans.

	**Cervical cord MRI**						**Brain MRI**			
**Cord level of ROI**	**C1–C3**		**C2/C3**		**C5–C7**		**C1–C3**		**C2/C3**	
**Site Nr/scanner vendor**	**Cord signal (median [min.–max.]**	**CNR_**UT**_ (Cord to CSF)**	**Cord signal (median [min.–max.]**	**CNR_**UT**_ (Cord to CSF)**	**Cord signal (median [min.–max.]**	**CNR_**UT**_ (Cord to CSF)**	**Cord signal (median [min.–max.]**	**CNR_**UT**_ (Cord to CSF)**	**Cord signal (median [min.–max.]**	**CNR_**UT**_ (Cord to CSF)**
1 Philips Intera	1,458 [1,437–1,460]	8.3	1,490 [1,371–1,499]	6.6	1,264 [1,205–1,270]	5.8^+^	1,367 [1,344–1,382]	4.5	1,226 [1,207–1,284]	5.1
2 Philips Acieva	1,459 [1,454–1,474]	9.4	1,407 [1,391–1,464]	7.4^+^	1,189 [1,146–1,2 05]	5.0	1,399 [1,377–1,416]	5.8^+^	1,268 [1,239–1,327]	5.4
3 Siemens TIM Trio	441 [423–445]	4.2	450 [449–471]	4.4	407 [405–417]	3.3	341 [324–341]	3.0	323 [320–334]	2.6
4 Philips Achieva	1,172 [1,162–1,191]	5.4	1,067 [1,050–1,150]	5.1	965 [956–978]	3.3	935 [925–942]	4.3	843 [784–845]	4.7
5 General Electric Discovery MR750	2,798 [2,387–2,810]	4.1	2,348 [2,052–2,501]	3.9	1,649 [1,344–1,889]	2.7*	2,444 [2,391–2,532]	2.3*	1,884 [1,853–1,971]	2.6
6 Siemens Prisma	296 [295–298]	4.4	266 [257–283]	3.4*	n.a.	n.a.	448 [443–454]	4.7	417 [404–418]	4.6

Cord signal within region of interest in arbitrary units (a.u.); CNR_UT_ (Cord to CSF): contrast-to-noise ratio per unit of time, expressed as the mean of three repeated MRIs; contrast-to-noise ratio per unit of time = (signal_cord_ - signal_CSF_)/mean (standard deviation of cord and CSF)* 1/sqrt(t), with t = acquisition duration of MRI.

*Significant pairwise differences between centers (Kruskal–Wallis tests with post-hoc pairwise comparisons using Bonferroni-corrected Dunn's tests):*

* *p < 0.050, +: reference center*.

*CSF, cerebrospinal fluid; min., minimum; max., maximum; n.a., not acquired*.

*Site Numbers: 1 = Ospedale San Raffaele, Milano; 2 = Ruhr-University of Bochum, Bochum; 3 = Hospital Universitari Vall d'Hebron, Barcelona; 4 = University College London, London; 5 = VU University Medical Center, Amsterdam; 6 = Basel University Hospital, Basel*.

### Cross-Sectional Area Quantification

As a first step, we compared the CSA results aggregated across all scanners at different measurement settings (software, cord level, cord, or brain MRI) according to a multicenter scenario with different scanner types. Two CSA estimates using SCT at the C2/3 level and three measurements at the C1–C7 level were excluded from the analyses due to erroneous segmentations and infolding artifacts, respectively (see section Similarities and Differences Between the Software Methods Regarding Partial Volume Effects and Handling of Cord Curvature).

#### Cross-Sectional Area Depends on the Cervical Cord Quantification Software Method

**Figure 3:**
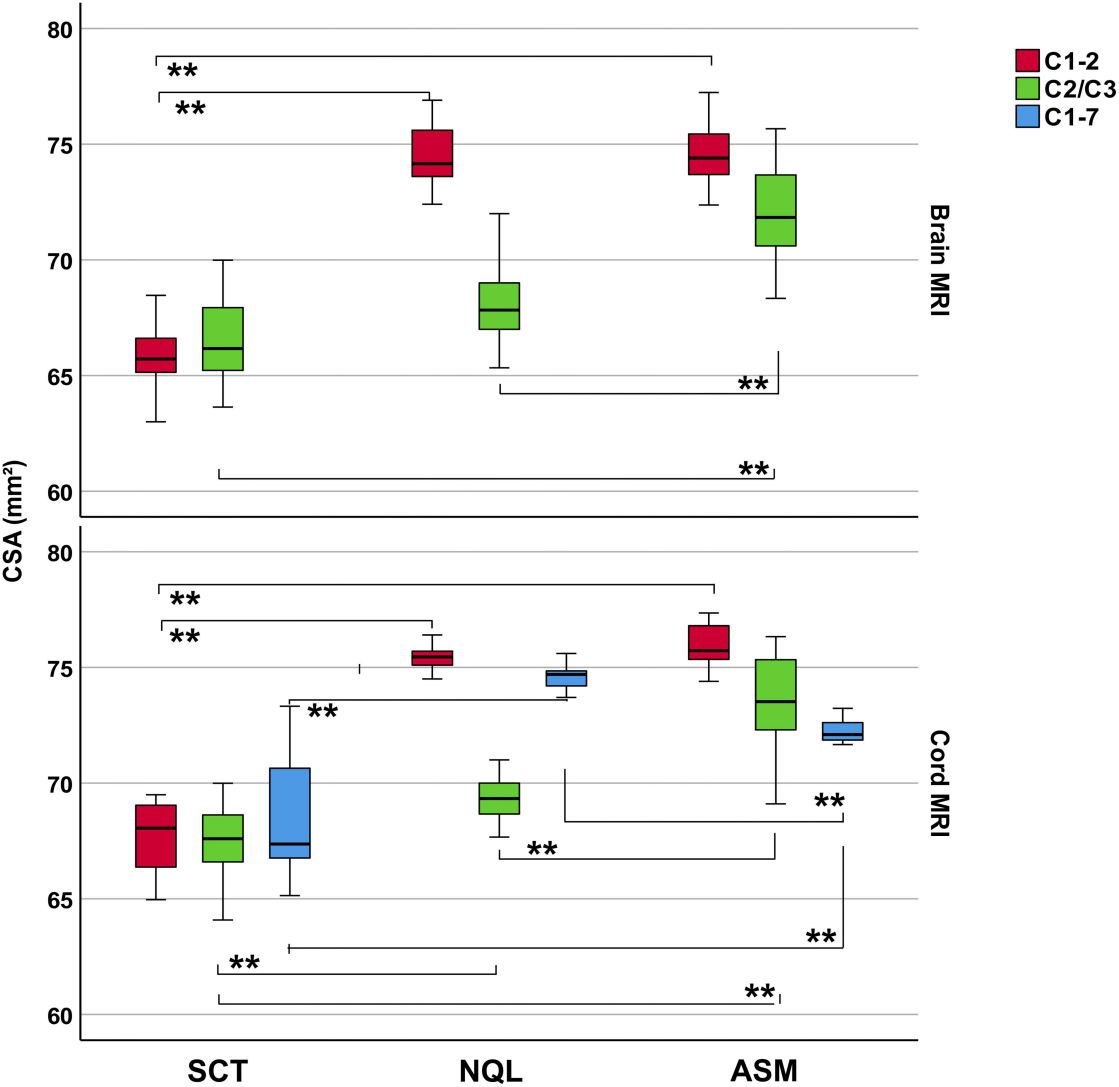
Cross-sectional area (CSA) estimates (pooled across all scanner sites and scan repetitions) differentiated for brain or cord MRI, software methods, and cord vertebral levels. Boxes: median and interquartile range, error bars: 95% interval. Significance between groups: Kruskal–Wallis test. *Post-hoc* pairwise comparisons including Dunn's *post-hoc* test with Bonferroni adjustment for multiple comparisons. **p < 0.001.

Table 3 shows mean and standard deviations of CSA (pooled across all centers) differentiated according to the software method at the different cord levels and to brain or cord MRI. Kruskal–Wallis tests between the software methods resulted for both brain and cord MRI in significantly lower CSA estimates using SCT for the C1–C2 cord level (brain MRI) and the C1–C2 and C1–C7 levels (cord MRI) when compared to those of both semiautomated techniques (all *p* < 0.001) (Figure 3). CSA results obtained with NQL and ASM were similar at the C1–C2 level (differences not significant), while at the C1–C7 level, CSA estimates obtained with ASM were lower than those when using NQL (*p* = 0.001), and CSA estimates at the C2/3 vertebral level were lower when using NQL than those when using ASM (*p* < 0.001).

**Table 3:** CSA estimates and inter-scanner repeatability of CSA across scanner sites using different software methods at different cord levels for brain or SC MRI.

**CSA Inter-scanner repeatability (6 scanners, 3 repeats)**						
	**Software method**	**Vertebral level**	***N***	**CSA** **Mean ± SD_**sites**_** **[mm^**2]**^**	**MMOD** **max. abs. Δ_scans_ [mm^**2**^]**	**CV** **[%]**
Cord MRI	SCT	C1–C2	18	67.63 ± 1.5	1.39	1.1
	NQL	C1–C2	18	75.35 ± 0.81	1.32	0.9
	ASM	C1–C2	18	75.96 ± 0.85	0.91	0.6
	SCT	C2/3	17	67.25 ± 1.97	1.87	1.6
	NQL	C2/3	18	69.43 ± 0.95	1.55	1.2
	ASM	C2/3	18	73.47 ± 2.1	3.44	2.6
	SCT	C1–C7	15	68.41 ± 2.55	1.27	1.0
	NQL	C1–C7	15	74.54 ± 0.6	**0.58**	**0.4**
	ASM	C1–C7	15	72.17 ± 0.64	**0.90**	**0.6**
Brain MRI	SCT	C1–C2	18	65.88 ± 1.77	1.98	1.6
	NQL	C1–C2	18	74.48 ± 1.3	1.07	0.8
	ASM	C1–C2	18	74.66 ± 1.34	1.01	0.7
	SCT	C2/3	17	66.56 ± 1.99	2.20	2.0
	NQL	C2/3	18	67.83 ± 2.16	3.11	2.6
	ASM	C2/3	18	72.08 ± 2.19	3.63	2.5

*CSA = mean ± SD averaged across all repetitions and all sites, avg. max. abs. Δ_scans_ = mean maximum observed difference (MMOD), **bold**: lowest CV and MMOD)*.

*CSA, cross-sectional area; MMOD, mean maximum observed difference; CV, coefficient of variation; SCT, Spinal Cord Toolbox; NQL, NeuroQLab; ASM, Active Surface Method*.

#### Inter-scanner Repeatability Depends on Cord Level, MRI Type, and Software

**Figure 4:**
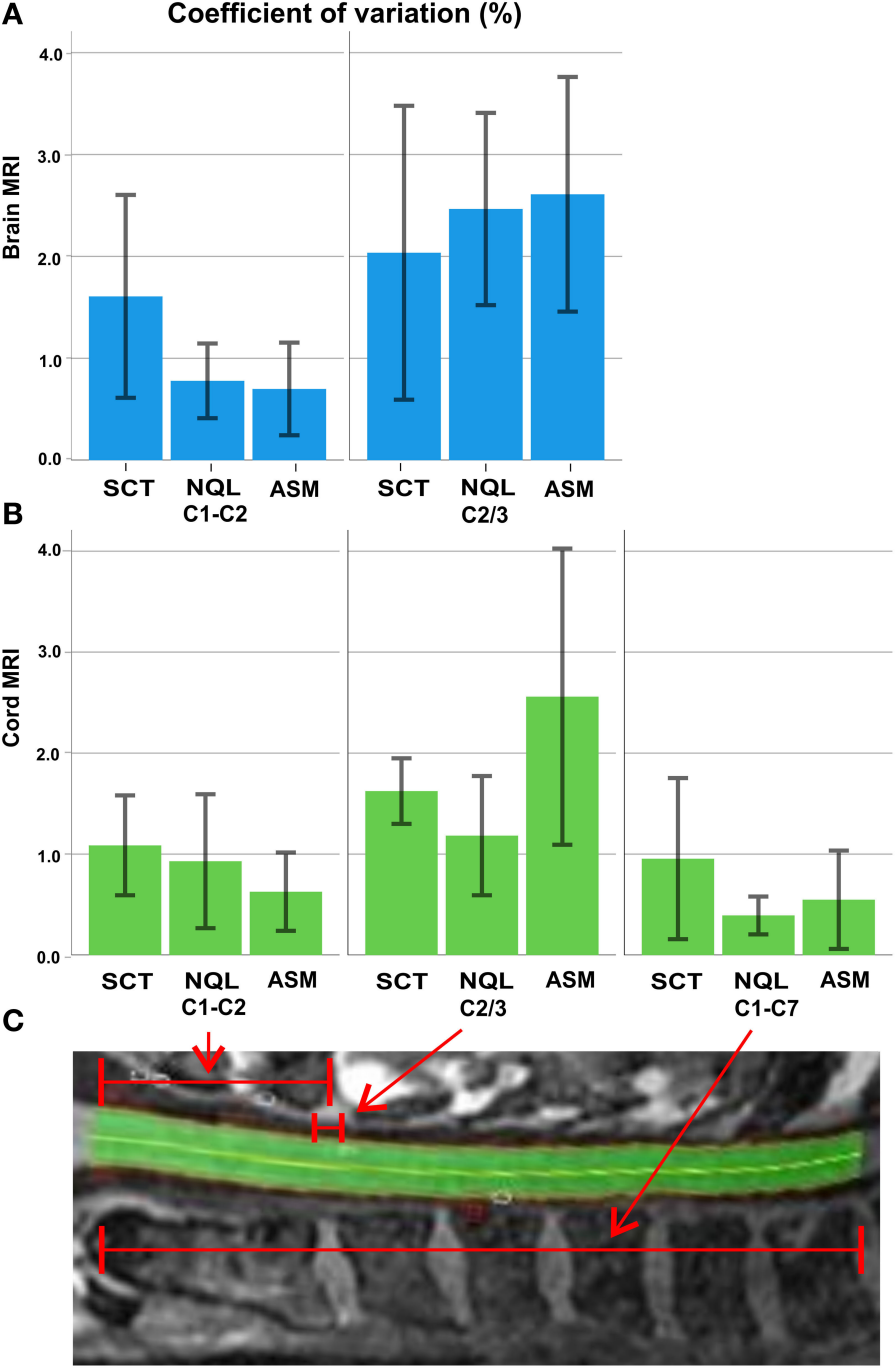
Overview of the agreement between the evaluation methods averaged across all sites: **(A)** coefficient of variation (%; error bars: ± 1 standard deviation) of the three software methods for brain MRI at the C1–C2 level and C2/3 level; **(B)** coefficient of variation (%) of the three software methods for cord MRI at the C1–C2, C2/3, and C1–C7 level; **(C)** illustration of the C1–C2, C2/3, and C1–C7 sections of the cervical cord (example: an NQL segmentation).

The repeatability of CSA across centers (inter-scanner) was best for NQL and ASM measured over the entire cervical cord (C1–C7) reflected by low CV of 0.4 and 0.6% and low MMOD of 0.6 and 0.9 mm^2^ (Table 3). Regarding the cord MRI acquisitions, the MMOD and CV across all software methods were only slightly higher at the C1–C2 vertebral levels compared to those at the C1–C7 level, while the CV and MMOD for measurements at the C2/3 interval were considerably worse than those at the C1–C7 level (Table 3, Figure 4).

The repeatability was similar for the semiautomatic methods (ASM and NQL) using brain or cord MRI at all levels, except for the C2/3 level in cord MRI, where ASM had a higher MMOD and CV than those for NQL. For the SCT method, the MMOD and CV were higher than those for NQL and ASM at the C1–C2 level and in the entire cervical cord. At the C2/3 level, the repeatability of SCT was superior to that of ASM using cord acquisitions and superior to that of both semiautomatic quantification techniques (NQL, ASM) using brain acquisitions (Table 3, Figure 4).

#### Comparing Cross-Sectional Area Results in Brain and Cervical Cord MRI

**Figure 5:**
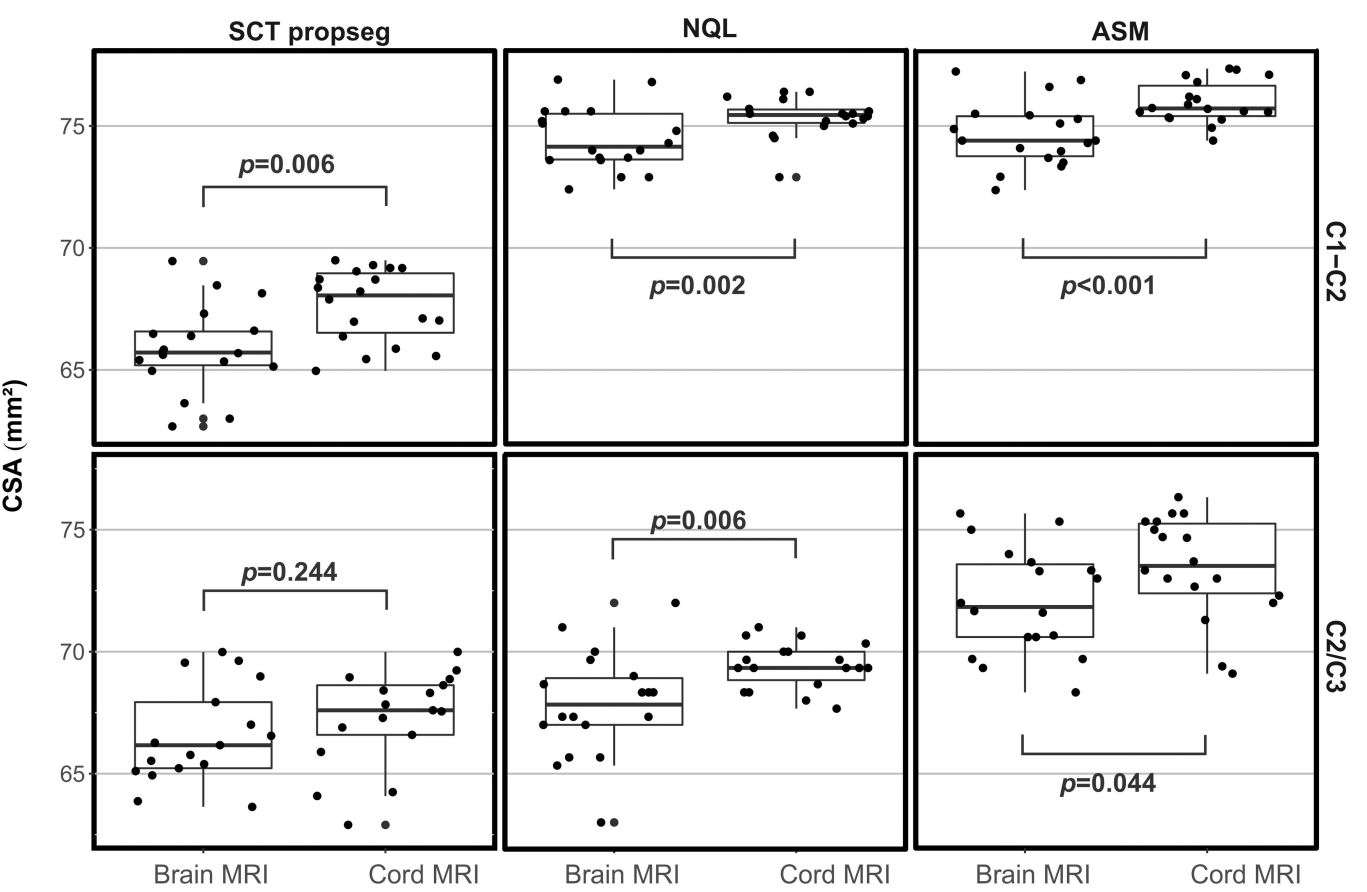
Cross-sectional area (CSA) across all centers based on brain or cord MRI using three different software methods at the C1–C2 or the C2/C3 vertebral level. Significance of group differences assessed using univariate ANOVAs with CSA as dependent variable and MRI acquisition type and scan repetition number as fixed factors.

CSA results based on brain acquisitions (aggregated across all centers) were significantly smaller than those from dedicated cord acquisitions at the same cord levels, except for the SCT method at the C2/3 level (Figure 5; CSA group differences assessed by Wilcoxon signed rank tests for paired samples). Still, the variability of CSA (expressed as CV) was not clearly higher when using the brain MRI scans than in dedicated cord scans (Table 3,Figure 4): the differences between brain and cord MRI of the CV of CSA were not statistically significant in any of the software methods at the C1–C2 or at the C2/3 level (all p > 0.050 using Wilcoxon signed rank tests for paired samples).

#### Scanner Dependencies

In a further analysis, we investigated the differences between CSA results of brain and cord MRI separately for each center. Table 4 shows CSA for brain and cord MRI at the C1–C2 and C2/C3 levels, estimated with the different software methods, differentiated with respect to scanner type. We observed different grades of deviation between brain and cord MRI results within and between centers (ranges: −4.4 to 0.4 mm^2^ at the C1–C2 level and −4.7 to 0.2 mm^2^ at the C2/3 level), with best agreement at both cord levels and for all software methods in the GE scanner (site nr. 5) and the Siemens scanner (site nr. 6), which both have a long magnet design (198 cm). In the combined results from all scanners, the mean differences between brain and cord scans ranged between −2.0 and −0.9 mm^2^ at the C1–C2 level and between −1.4 and −0.8 mm^2^ at the C2/C3 level, depending on the software method used. Specifically, the brain–cord acquisition differences at the C1–C2 level seemed larger when using SCT compared to those when using NQL or ASM, while they were smaller for SCT compared to those for ASM and NQL at the C2/3 level.

**Table 4:** Comparison of CSA results for brain and SC MRI differentiated by center.

**Cord level**			**C1–C2**										**C2/C3**								
**Software method**				**SCT**			**NQL**			**ASM**			**SCT**			**NQL**			**ASM**		
**Site Nr**.	**Ven-dor**	**Magnet-length (cm)**	***N***	**CSA (mm** ^**2**^ **, mean)**		**Diff**.	**CSA (mm** ^**2**^ **, mean)**		**Diff**.	**CSA (mm** ^**2**^ **, mean)**		**Diff**.	**CSA (mm** ^**2**^ **, mean)**		**Diff**.	**CSA (mm** ^**2**^ **, mean)**		**Diff**.	**CSA (mm** ^**2**^ **, mean)**		**Diff**.
				**Brain**	**Cord**		**Brain**	**Cord**		**Brain**	**Cord**		**Brain**	**Cord**		**Brain**	**Cord**		**Brain**	**Cord**	
1	PHI	157	3	65.9	69.0	−3.1	74.8	75.4	−0.6	74.9	75.1	−0.2	66.0	68.8	−2.8	69.3	69.9	−0.6	73.3	73.1	0.2
2	PHI	157	3	65.5	68.8	−3.3	73.8	75.1	−1.3	73.8	75.5	−1.7	67.3	68.2	−0.9	67.3	68.6	−1.3	71.9	73.2	−1.3
3	SIE	198	3	65.0	67.8	−2.8	73.2	75.1	−1.9	73.6	75.8	−2.2	64.9	68.7	−3.8	65.8	68.8	−3	69.6	74.3	−4.7
4	PHI	157	3	64.1	68.5	−4.4	73.4	74.8	−1.4	73.5	76.4	−2.9	66.2	67.9	−1.7	66.1	69.6	−3.5	71.5	74.2	−2.7
5	GE	194	3	66.1	65.9	0.2	75.3	75.7	−0.4	75.3	75.6	−0.3	65.8	63.7	2.1	68.8	69.9	−1.1	72.8	71.4	1.4
6	SIE	198	3	68.7	67.0	1.7	76.4	76.0	0.4	76.9	77.3	−0.4	69.5	67.0	2.5	69.7	69.9	−0.2	73.5	74.5	−1
**All sites**			**18**	**65.9**	**67.8**	−2.0^*^	**74.5**	**75.4**	−0.9^*^	**74.7**	**76.0**	−1.3^*^	**66.7**	**67.4**	**−0.8**	**67.8**	**69.4**	−1.6^*^	**72.1**	**73.5**	−1.4^*^

*PHI, Philips; SIE, Siemens; GE, General Electric; SCT, Spinal Cord Toolbox; NQL, NeuroQLab; ASM, Active Surface Method; CSA, cross-sectional area; Diff, difference between mean of brain and cord MRI results [mm^2^]*.

*Marked bold: results of CSA pooled across all sites;*

* *p < 0.050 using paired Kruskal–Wallis test*.

*Site Numbers: 1 = Ospedale San Raffaele, Milano; 2 = Ruhr-University of Bochum, Bochum; 3 = Hospital Universitari Vall d'Hebron, Barcelona; 4 = University College London, London; 5 = VU University Medical Center, Amsterdam; 6 = Basel University Hospital Basel, Basel*.

#### Consistency of Cross-Sectional Area Results Between the Centers

**Figure 6:**
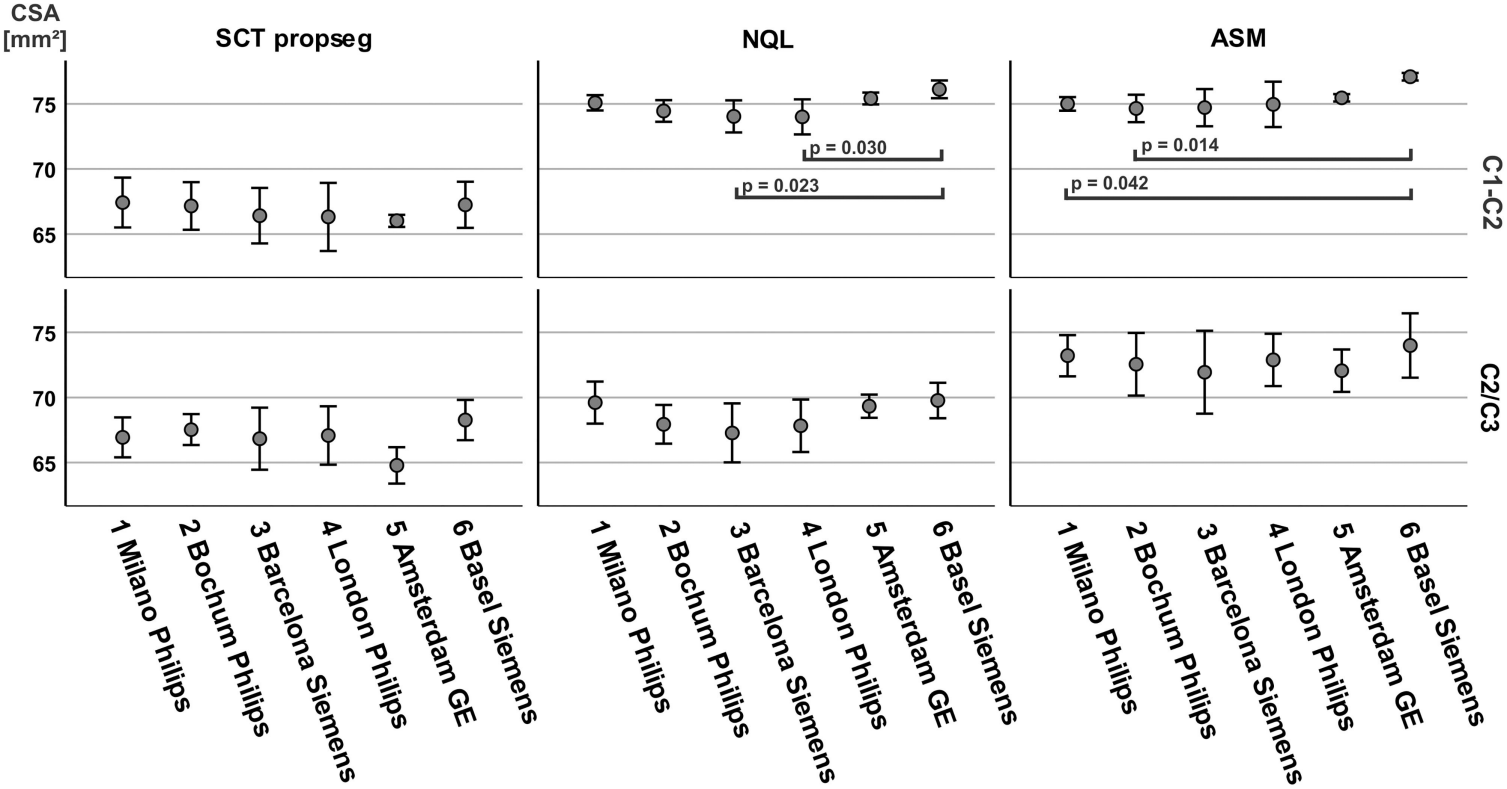
Between-center comparison of cross-sectional area (CSA), differentiated for post-processing software and cord level. CSA was aggregated across brain and cord MRI (*N* = 6, for each center). Significance between groups by Kruskal–Wallis test with *post-hoc* comparisons between pairs of sites adjusted for multiple comparisons with Dunn's *post-hoc* test and Bonferroni adjustment.

Between-center agreement of CSA at the C1–C2 or C2/3 cord levels was assessed separately for each software method using Kruskal–Wallis test with *post-hoc* comparisons between pairs of sites adjusted for multiple comparisons by aggregating measurements of brain and cord MRI (*N* = 6 measurements at each center). The results are depicted in Figure 6. At the C1–C2 cord level, we observed significantly higher CSA in site 6 compared to those in site 3 and site 4 when using NQL and also compared to those in center 1 and center 2 for the ASM method. There were no other center differences (all *p* > 0.050) for ASM and NQL at the C1–C2 level. Overall, there were no significant inter-center differences for SCT at both cord levels and for NQL and ASM at the C2/3 cord level.

## Discussion

Quantification of CSA has gained increasing attention over the past years, and techniques assessing CSA have improved in terms of robustness and reproducibility. However, to be successfully established in multicentric MS studies, CSA quantification still lacks harmonized procedures, e.g., agreement on a common vertebra level region to be measured.

In the present traveling volunteer study, we studied three popular fully automated and semiautomated techniques for CSA assessment (SCT, NQL, ASM) at different cervical cord regions derived from brain and SC scans of a single healthy volunteer scanned at six different European MS centers in order to propose a common cord level for reliable CSA assessment. Our results were in agreement with the findings of recent studies that showed good concordance between results of brain and SC MRI using the NQL method in the upper cervical cord and extended these assessments by including other software tools and SC levels and a larger number of different scanners (15, 22).

The CSA results were dependent on the software used and, as expected, on the cord region included in the evaluation. Repeatability, especially across centers, was best when scanning the entire cervical cord. Agreement was better between similar types of approaches (e.g., semiautomated) than between different types (e.g., semiautomated and fully automated) techniques, as we observed lower CSA values when using a fully automated approach regardless of the vertebra level used.

### Absolute Cross-Sectional Area Results Depend on the Evaluation Software and Cord Level

In concordance with a recent study (11), absolute results of the automatic segmentation with SCT (PropSeg) at the upper portion of the cervical cord were systematically lower than CSA results assessed by the semiautomated methods ASM or NQL. In the present study, we show as a new finding that the mean CSA results when obtained from the entire cervical cord were also significantly lower when using SCT than those when using ASM or NQL. Those systematic differences between SCT and the semiautomated methods, independent of the specific cervical region, are probably due to different ways the algorithms define the contour of the cord and assign voxels at the edges of the cord as belonging or not belonging to the cord. Since ASM and NQL take partial volume effects into account, while SCT does not, these differences between methods could be the reason for the overall consistency between CSA derived from NQL and ASM: the CSA estimates of NQL and ASM were in good agreement when assessing the C1–C2 vertebral level, while mean CSA results in the entire cervical cord assessed using ASM were lower than the NQL results.

Since CSA varies in the cranio–caudal direction across the cervical SC, the exact choice of the cord level has a major influence on the absolute CSA results (Figure 4C) (16, 33). Obviously, calculation of mean CSA over different segments involves averaging some of that variation, so subtle differences between cord levels may be lost, although noise is reduced as measurement takes place across a larger volume. Additionally, the segmentation quality of the methods might differ in their sensitivity to image degradation in the caudal part of the cervical cord due to signal drop off (Table 2), potential geometrical image distortions, or the presence of emerging nerve roots. Given the high reproducibility of the NQL and ASM results at the C1–C2 and C1–C7 levels, the differences in the absolute results when quantifying the entire cervical cord might be due to different susceptibilities of the methods to those specific pitfalls mentioned above when segmenting a long cord section. Additionally, effects of the cervical cord curvature, which is more pronounced for the entire cervical cord than in the smaller upper cervical cord section, are accounted for in the ASM method, but not in NQL. Thus, the CSA results in NQL might be slightly overestimated when measuring the entire cervical cord due to varying volume contributions at the upper and lower boundary of the cord section. Furthermore, the NQL method seemed less suited than ASM or SCT to quantify the single 3-mm cord section at the C2/3 level, where NQL measured considerably lower CSA values compared to ASM method using both brain and cord MRI (Figure 3, Table 3). NQL uses an intensity-based Gaussian mixture model for automatic tissue classification of the cord and the surrounding CSF, which typically requires a large number of voxels and hence might lose precision when applied to very small volumes like the 3-mm section at the C2/3 level that was investigated in the present study.

### Comparing Repeatability Between the Software Methods and Cord Levels

In the multicentric analysis across the six centers, the repeatability was best when assessing the entire cervical cord (CV ≤ 1.0% for all software methods) and slightly worse in the upper portion of the cervical cord (Table 3). Furthermore, in these cord sections, the variability of the results was higher when using the SCT method (CV ≤ 1.6%) than those when using the NQL (CV ≤ 0.9%) or ASM (CV ≤ 0.7%), reflecting a reduced repeatability of the automated software method compared to the semiautomated segmentations in these measurement settings.

Segmentation of the C2/3 cord level regarding a small 3-mm cord section [comparable to the classical method proposed by Losseff et al. (6)] led to marked increases of the variability in all methods compared to measurements at the C1–C2 or C1–C7 levels (Table 3, Figures 3A,B). While segmentation of small cord sections might theoretically be advantageous when assessing local changes, its sensitivity to image inhomogeneity and partial volume effects probably leads to marked variability of the results, thus making this method less feasible for use in larger studies. Nevertheless, the SCT software method seemed to be more robust when looking at a very small cord section (CV ≤ 2.0%) than NQL or ASM (CV ≤ 2.6 and ≤ 2.5%, respectively).

In addition to these cord section-dependent effects, the variability of CSA values in a multicentric analysis can partly result from different image qualities between the centers, as reflected by differences of the cord-to-CSF contrast (Table 2). Despite that the consensus on MRI protocols used in the network of the participating centers was aimed at homogeneous spatial resolution and contrast features for brain and SC MRI, subtle differences between the sequence details related to scanners and different vendors remained. Enhanced cord to CSF CNR in cord MRI achieved by certain vendors compared to others can be due to differences in echo time (TE), inversion time (TI), and repetition time (TR) protocol settings, but also in scanner and coil design (Table 2, Figure 2). Such scanner-dependent contrast differences between cord and surrounding CSF could lead to changes in the partial volume effects at the boundary of the SC, with different effects on the segmentation results of the software methods. Accordingly, we found varying CSA differences between pairs of scanners, which differed between the three software methods (Figure 6). These findings underline the need for careful protocol and contrast harmonization between centers in multicentric studies, since inter-center differences based on different scanners and protocols seem to contribute to the limitations of detectability of disease-related CSA changes.

### Comparing Brain and Spinal Cord MRI

Comparison between brain and cord CSA estimates at the same levels showed for all software methods used slightly lower CSA when using brain MRI. Different factors can contribute to differences in the CSA results of brain and SC MRI: one is based on gradient non-linearity distortions that may become relevant at the edges of the magnet especially in modern short-bore scanners. This may particularly influence the CSA quantification based on brain MRI, since the upper cervical cord is located off-center in the sagittal images, at the periphery of the field of view. A thorough analysis of these effects and possible ways of compensating or avoiding their impact on CSA measurements has recently been published (23), which showed that non-linear gradient distortions will lead to lower CSA results when quantifying using brain MRI. Our results of lower brain MRI CSA results compared to cord MRI confirmed these findings (Figure 5, Table 4). Scanners with long-bore magnets should be less susceptible to these effects. Different grades of deviations between brain and SC results between the sites might partly be due to different magnet types and gradient systems provided by the different vendors.

### Limitations

This study considered only one healthy traveling volunteer. A limitation of the study results is given by reduced SC MRI image quality due to infolding artifacts in one of the centers, leading to a slightly reduced statistical power of the SC MRI evaluations. Furthermore, it would have been preferable to have more than one healthy participant in this reproducibility study.

The comparability of the results and the reproducibility in the different measurement constellations could have been further improved if procedures were used to ensure that the evaluation takes place in exactly the same regions (for example, by transferring binary segmentation masks from one method to the other). In this study, we have limited the evaluations to using the individual methods “as is” and to aligning the regions only on the basis of anatomical markers. We think that this corresponds more to the typical situation in larger studies where a decision has to be made for a certain method.

Furthermore, stricter standardization of MRI protocols than applied in this study would probably lead to a reduction in variability of CSA between scanners in multicentric studies. Recently, a fully harmonized examination protocol for different scanner vendors, including sagittal 3D T1w imaging and other sequences for quantitative examination of the SC was freely made available to the research community (the *spine generic protocol*, https://spinalcordmri.org/protocols). This generic SC protocol has successfully been implemented in 42 MRI centers worldwide in order to generate a harmonized multi-subject dataset (34). Future multicentric studies on CSA quantification should adapt to this approach.

### Conclusions and Recommendations for the “Optimal” Cervical Cord Section and Software Method for Cross-Sectional Area Assessment

Aiming at optimal reproducibility of the compared methods, dedicated isotropic SC MRI using 3D acquisition should be acquired whenever possible, since repeatability was best when scanning the entire cervical cord (Table 5). Nevertheless, CSA quantification of only the upper part of the cervical cord, even based on brain MRI, seemed to entail only minor loss of repeatability and comes with major advances in terms of acquisition time and patient comfort. Thus, if lengthy brain imaging protocols are used and the additional acquisition of dedicated SC MRI is not feasible, CSA quantification of the C1–C2 cord level making use of sagittal 3D T1w brain MR (using a combined head and neck coil) can be used to achieve reliable CSA results.

Table 5Recommendations for cervical cord section and software method in CSA assessment.

**Table:** 

**CSA quantification**	**Recommendation**	**Alternative**
Which MRI acquisition?	Dedicated cervical cord MRI incl. isotropic 3D T1w Pro: best CSA repeatability Con: long duration of brain and cervical cord MRI	brain MRI incl. sagittal 3D T1w to cover the upper cervical cord Pro: Patient comfort/shorter MRI duration; only minor loss of CSA repeatability in brain MRI at C1–C2 Con: limited to upper cervical cord levels
Which vertebral level?	Entire cervical cord (C1–C7) Pro: best CSA repeatability Con: averaging of regional changes	Regional cervical cord sections (e.g., C1–C2) Con: repeatability of CSA slightly reduced

**Table:** 

**Which software?**	**ASM**	**NQL**	**SCT**
Consider number of study participants	Semiautomated	Semiautomated	Fully automated
Availability	Commercial license for research purposes	Free research license upon request	Free license for research (open source)
	Pro: high repeatability of CSA Con: time-consuming for raters in large datasets Con: license available with costs	Pro: high repeatability of CSA Con: time-consuming for raters in large datasets	Con: repeatability slightly reduced Pro: suited for automatic evaluation in large datasets Pro: freely available
Comparability between methods?	CSA differs between software and cord levels		

Looking at disease-related changes in the cervical cord, which was not part of the present study, may lead to a different point of view. Quantification involving the entire cervical cord means averaging over processes that may be focused to certain cord regions, and good reproducibility may thus be traded off for less sensitivity to subtle changes. Recent studies have shown that cord atrophy in MS especially involves the upper cervical cord level (7, 15, 25, 26, 35), so CSA quantification in the upper portion of the cervical cord, involving only a smaller cord interval, may be advantageous in clinical studies of MS patients. Recently, differences in the local patterns of cervical cord atrophy between the relapsing-remitting MS types and progressive forms have been shown, pointing to increasing involvement of the caudal cervical cord levels in the secondary progressive and primary progressive types of MS (10). As a consequence, the cord level for CSA evaluation in MS studies should be optimized with regard to the subtypes of MS patients.

Comparing the different software methods, the semiautomated methods NQL and ASM seemed to be similarly suitable and robust and be superior compared to SCT with regard to reproducibility. All three software methods performed similarly on brain and SC acquisitions. When analyzing large patient studies, the automated SCT method can be clearly advantageous with regard to analysis time (the semiautomated methods both take about 5 to 7 min for processing and handling a single dataset). The choice of the optimal method may depend on the number of patients included in the study. Furthermore, the use of SCT may be beneficial because it is freely available for scientific purposes, while NQL can be used upon request to Mevis, and ASM is distributed with costs.

Since the absolute CSA results of the different software methods and the different cord levels deviate considerably from each other, it is important to keep the acquisition and post-processing methodology identical within a study and to report these study details in publications. When comparing CSA results of different publications, absolute results should be regarded carefully, considering different evaluation methods. Longitudinal rates of change of CSA might be less sensitive to these methodological effects. On the other hand, longitudinal analyses can also entail specific problems that may affect the accuracy of CSA measurements. For example, the quality of patient repositioning or possible hardware or software changes between follow-ups have to be taken into account.

Center-dependent effects that have been detected in this traveling-volunteer reproducibility study have to be considered when pooling data from different centers. Differences in scanner geometry, coil design, and variability of image contrast have effects on CSA estimates and thereby limit the sensitivity to detect small disease-related cervical cord changes in multicentric studies.

Further longitudinal studies on MS patients and healthy controls to be acquired at different centers are warranted to further optimize cord levels and software tools for CSA quantification in multicentric studies.

## Data Availability Statement

The datasets analyzed for this study are freely available for scientific research upon request to MAGNIMS (https://www.magnims.eu/magnims-cord-dataset).

## Ethics Statement

The study involving human participants was conducted in line with the International Conference on Harmonization Good Clinical Practice (ICH GCP) and was reviewed and approved by the local ethics boards of the involved institutions. At each site, the MRI acquisitions reported in the present study were conducted after signed informed consent of the participant, who gave their written informed consent to take part in the repeated MRI acquisitions at different centers, both for the use of the anonymized MRI data for scientific purposes within the scope of the present study and for sharing the data with the research community. The institutional review boards were 1) Ospedale San Raffaele; Milano, Italy: Ethics Review Board of IRCCS San Raffaele Scientific Institute, Milan, Italy. 2) St. Josef Hospital, Ruhr-University of Bochum; Bochum; Germany: Ethics Commision of the Medical Faculty of the Ruhr-University Bochum, Bochum, Germany. 3) Hospital Universitari Vall d'Hebron Barcelona; Spain: Research Ethics Committee (REC) of the University Hospital Vall d'Hebron, Barcelona, Spain. 4) University College London; London; Great Britain: London - Harrow Research Ethics Committee (REC), London, Great Britain. 5) VU University Medical Center; Amsterdam; The Netherlands: Medical Ethics Review Board of the Amsterdam UMC University Medical Centers (METc, Medisch Ethische Toetsingscommissie), Amsterdam, The Netherlands. 6) Basel University Hospital; Basel; Switzerland: Ethics Committee Northwest and Central Switzerland (Ethikkommission Nordwest- und Zentralschweiz (EKNZ), Basel, Switzerland).

## Author Contributions

CL, HV, and FB: conceptualization. CL, HV, IB, ÀR, MR, and FP: methodology. CL, BB, IB, FP, and MR: software. CL, BB, and HV: formal analysis and writing–original draft. CL, PV, KP, ÀR, DP, and MY: investigation. CL, HV, IB, FB, LK, ÀR, MF, and CG: resources. CL, FP, JS-G, CG, and HV: data curation. CL, BB, FP, PV, KP, IB, DP, ÀR, JS-G, CG, LK, MR, MF, MY, FB, and HV: writing–review & editing. CL, BB, HV, and IB: visualization. LK, ÀR, FB, MF, CL, and MR: supervision. CL: project administration. All authors contributed to the article and approved the submitted version.

## The Magnims Study Group

The members of the MAGNIMS Study Group Steering Committee are: F. Barkhof and H. Vrenken, VU University Medical Center, Amsterdam, Netherlands; O. Ciccarelli and T. Yousry, Queen Square MS Center, UCL Institute of Neurology, London, UK; N. De Stefano, University of Siena, Siena, Italy; C. Enzinger, Department of Neurology, Medical University of Graz, Graz, Austria; M. Filippi and M. A. Rocca, IRCCS San Raffaele Scientific Institute, Vita-Salute San Raffaele University, Milan, Italy; C. Gasperini, San Camillo-Forlanini Hospital, Rome, Italy; L. Kappos, University of Basel, Basel, Switzerland; J. Palace, University of Oxford Hospitals Trust, Oxford, UK; A. Rovira and J. Sastre-Garriga, Hospital Universitari Vall d'Hebron, Universitat Aut‘onoma de Barcelona, Barcelona, Spain.

## Conflict of Interest

CL received a research grant by the German Federal Ministry for Education and Research, BMBF, German Competence Network Multiple Sclerosis (KKNMS), grant no. 01GI1601I, has received consulting and speaker's honoraria from Biogen, Bayer, Daiichi Sanykyo, Merck Serono, Novartis, Sanofi, Genzyme and TEVA. BB reports financial support by the German Federal Ministry for Education and Research, BMBF, German Competence Network Multiple Sclerosis (KKNMS), grant no. 01GI1601I. FP has received grants from MRC and the NIHR biomedical research center at UCLH, outside the submitted work. PV reports speaker honoraria from Biogen and ExceMed, outside the submitted work. IB is supported by research grants from Teva Pharmaceuticals, Merck Serono, Novartis Pharma and the Dutch MS Foundation (grants to HV and FB), outside the submitted work. DP has received honoraria as speaker from Novartis and Genzyme, and a grant by Biogen, outside the submitted work. ÀR reports personal fees from Novartis, Sanofi-Genzyme, Bayer, Roche, Biogen, Neurodiem, Bracco, Merck Serono, Teva Pharmaceuticals, and Icometrix; and non-financial support from SyntheticMR, outside the submitted work. JS-G reports grants and personal fees from Sanofi Genzyme; and personal fees from Almirall, Biogen, Celgene, Merck Serono, Novartis, Roche, and Orchid Pharma and Biopass, outside the submitted work; and is member of the Editorial Committee of Multiple Sclerosis Journal and director of the Scientifi Committee of Revista de Neurologia. CG receives research grants outside the submitted work from ISRT, EPSRC, Wings for Life, UK MS Society, Horizon2020, Biogen and Novartis; is editorial board member of Functional Neurology. LK reports personal fees from Actelion, Addex, Biotica, Celgene Receptos, Sanof Genzyme, Eli Lilly, Mitsubishi, Ono Pharmaceutical, Pfier, Sanofi Santhera Pharmaceuticals, Siemens, Teva Pharmaceuticals, UCB, and XenoPort; grants and personal fees from Bayer HealthCare Pharmaceuticals, Biogen, Merck Serono, and Novartis; and grants from F Hoffann-La Roche, EU, Innoswiss, Roche Research Foundation, Swiss Multiple Sclerosis Society, and Swiss National Research Foundation, outside the submitted work. MR reports personal fees from Biogen, Novartis, Sanofi Genzyme, Teva Pharmaceuticals, Merck Serono, and Roche, outside the submitted work; and receives research support from the Italian Ministry of Health and Fondazione Italiana Sclerosi Multipla. MF reports personal fees from Biogen, Merck Serono, Novartis, and Teva Pharmaceuticals, outside the submitted work; receives research support from Biogen, Merck Serono, Novartis, Teva Pharmaceuticals, Roche, Italian Ministry of Health, Fondazione Italiana Sclerosi Multipla, and Fondazione Italiana di Ricerca per la SLA; and is editor-in-chief of the Journal of Neurology. FB received, outside the submitted work, compensation for consulting services by Bayer, Biogen; receives research support from Biogen, GE Healthcare, IMI-EU IXICO Ltd., Merck-Serono, Roche, The Dutch Foundation MS Reserach; he is Editor in chief of “Clinical Neuroradiology - The ESNR textbook”. HV reports grants and consulting fees paid directly to their institution from Merck Serono and Novartis, and grants from Teva Pharmaceuticals, outside the submitted work. The remaining authors declare that the research was conducted in the absence of any commercial or financial relationships that could be construed as a potential conflict of interest.

## Publisher's Note

All claims expressed in this article are solely those of the authors and do not necessarily represent those of their affiliated organizations, or those of the publisher, the editors and the reviewers. Any product that may be evaluated in this article, or claim that may be made by its manufacturer, is not guaranteed or endorsed by the publisher.
