# Supplementary material for: Quantification of Cervical Cord Cross-Sectional Area: Which Acquisition, Vertebra Level, and Analysis Software? A Multicenter Repeatability Study on a Traveling Healthy Volunteer
Source: Front Neurol. 2021 Aug 4;12:693333. doi: 10.3389/fneur.2021.693333 (PMC8371197; doi:10.3389/fneur.2021.693333)

Supplement to:

Quantification of Cervical Cord Cross-Sectional Area: Which Acquisition, Vertebra Level and Analysis Software? A Multicenter Repeatability Study on a Traveling Healthy Volunteer

C. Lukas[1*] et al., 2021

*** Correspondence:**
Carsten Lukas, Institute of Neuroradiology, Ruhr-University Bochum / St. Josef Hospital, Gudrunstrasse 56, D-44791 Bochum, Germany
carsten.lukas@ruhr-uni-bochum.de

Figure S1
Placement of ROIs for determination of the contrast to noise ratio between cord and CSF at the vertebral levels: C1-C2 (ROI cord: 1, CSF: 3), C2/3 (ROI cord: 2; CSF 4) and the C5-C7 (ROI cord: 6; CSF: 7 & 8).
.

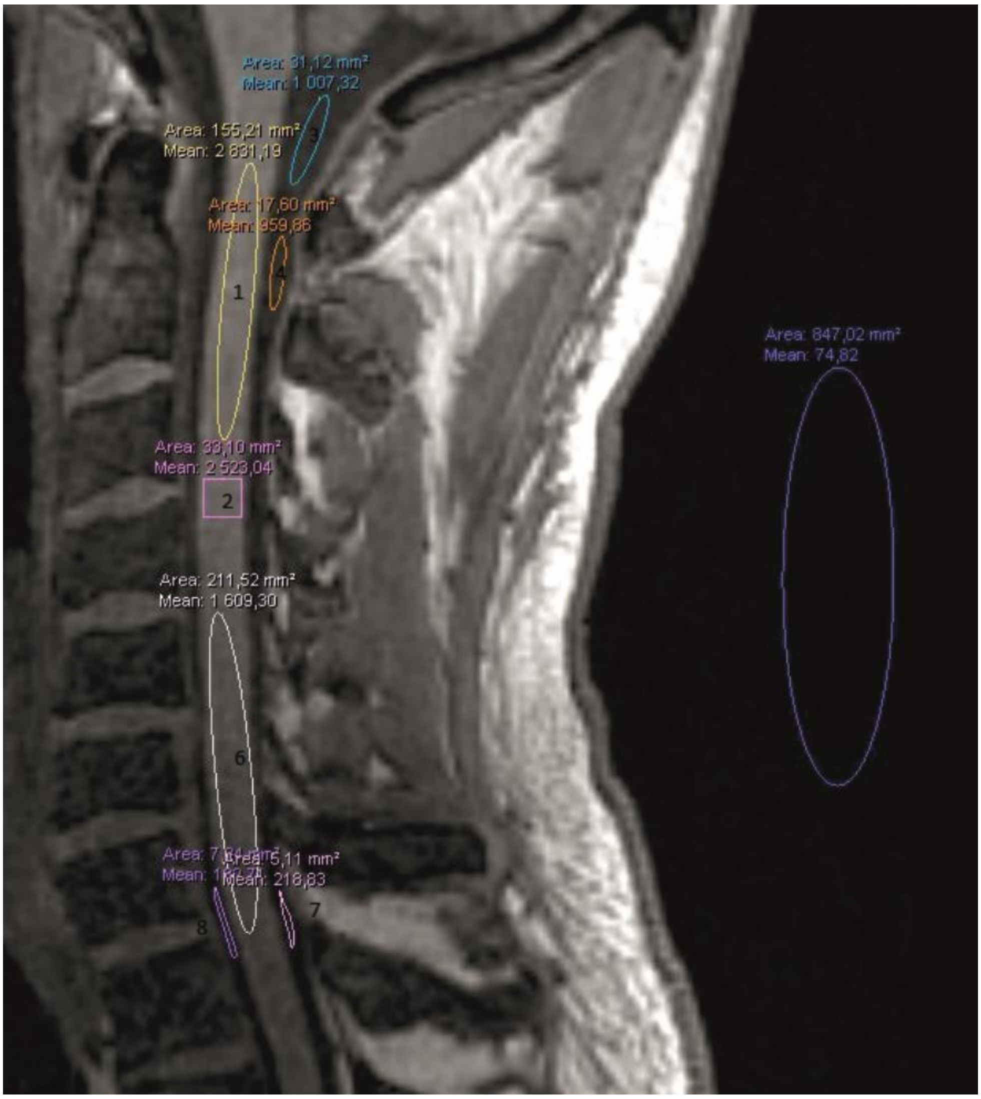

Supplement: Supplementary file 1 [file Table_1.pdf]
